# Resting-state electroencephalography changes in poststroke patients with visuospatial neglect

**DOI:** 10.3389/fnins.2022.974712

**Published:** 2022-08-10

**Authors:** Yichen Zhang, Linlin Ye, Lei Cao, Weiqun Song

**Affiliations:** Department of Rehabilitation, Xuanwu Hospital, Capital Medical University, Beijing, China

**Keywords:** stroke, visuospatial neglect, resting-state EEG, delta/alpha ratio, alpha oscillation

## Abstract

**Background:**

This study aimed to explore the electrophysiological characteristics of resting-state electroencephalography (rsEEG) in patients with visuospatial neglect (VSN) after stroke.

**Methods:**

A total of 44 first-event sub-acute strokes after right hemisphere damage (26 with VSN and 18 without VSN) were included. Besides, 18 age-matched healthy participants were used as healthy controls. The resting-state electroencephalography (EEG) of 64 electrodes was recorded to obtain the power of the spectral density of different frequency bands. The global delta/alpha ratio (DAR), DAR over the affected hemispheres (DAR_AH_), DAR over the unaffected hemispheres (DAR_UH_), and the pairwise-derived brain symmetry index (pdBSI; global and four bands) were compared between groups and receiver operating characteristic (ROC) curve analysis was conducted. The Barthel index (BI), Fugl-Meyer motor function assessment (FMA), and Berg balance scale (BBS) were used to assess the functional state of patients. Visuospatial neglect was assessed using a battery of standardized tests.

**Results:**

We found that patients with VSN performed poorly compared with those without VSN. Analysis of rsEEG revealed increased delta and theta power and decreased alpha and beta power in stroke patients with VSN. Compared to healthy controls and poststroke non-VSN patients, patients with VSN showed a higher DAR (*P* < 0.001), which was significantly positively correlated with the BBS (DAR: *r* = –0.522, *P* = 0.006; DAR_AH_: *r* = –0.521, *P* = 0.006; DAR_UH_: *r* = –0.494, *P* = 0.01). The line bisection task was positively correlated with DAR (*r* = 0.458, *P* = 0.019) and DAR_AH_ (*r* = 0.483, *P* = 0.012), while the star cancellation task was only positively correlated with DAR_AH_ (*r* = 0.428, *P* = 0.029). DAR_AH_ had the best discriminating value between VSN and non-VSN, with an area under the curve (AUC) of 0.865. Patients with VSN showed decreased alpha power in the parietal and occipital areas of the right hemisphere. A higher parieto-occipital pdBSI_alpha_ was associated with a worse line bisection task (*r* = 0.442, *P* = 0.024).

**Conclusion:**

rsEEG may be a useful tool for screening for stroke patients with visuospatial neglect, and DAR and parieto-occipital pdBSI_alpha_ may be useful biomarkers for visuospatial neglect after stroke.

## Introduction

Visuospatial neglect (VSN) is the most frequent neglect syndrome, characterized by failure to orient or respond to visual stimuli presented in the contralesional space (Heilman and Valenstein, 1979; Parton et al., 2004), particularly in patients with right hemisphere damage (Ten Brink et al., 2017). The prevalence of VSN after unilateral stroke is 30% (Esposito et al., 2020), and it disrupts basic activities of daily living (such as dressing and walking; Nijboer et al., 2013; Aravind and Lamontagne, 2018) and increases the risk of falls (Chen et al., 2015). Worsely, many individuals with VSN are unaware of these deficits. Furthermore, VSN hinders the ability to perform rehabilitation and limits recovery during early post-stroke neuroplasticity enhancement. VSN is an important predictor of poor functional recovery 1 year after stroke (Jehkonen et al., 2000; Hammerbeck et al., 2019). Despite its high prevalence and serious sequelae, VSN often remains unrecognized and undertreated because of the heterogeneity of its clinical manifestations and the limitations of assessment methods (Puig-Pijoan et al., 2018). Pencil-and-paper clinical tests are the most commonly used tools to assess VSN, but they sometimes lack sensitivity (Azouvi, 2017). Some patients with severe VSN symptoms may be unable to complete the scale assessment, whereas patients with mild VSN symptoms may have normal scale assessments. Therefore, in addition to assessing the severity of VSN symptoms from a behavioral perspective, new assessment tools that can sensitively reflect dynamic changes at physiological levels are needed.

Electroencephalography (EEG) is a non-invasive method with high temporal resolution, which contributes to the rapid evaluation of instantaneous brain function. Closed-eye resting-state EEG (rsEEG) provides an important opportunity to examine EEG oscillatory patterns of spontaneous brain activity unbiased by any task (Fingelkurts and Fingelkurts, 2015). Specific EEG oscillation patterns are associated with specific psychological or behavioral states (Herrmann et al., 2016). A previous study showed that alpha desynchronization in the spatially contralateral hemisphere of attention is a reliable marker of attentional orientation in the healthy human brain (Lasaponara et al., 2019). A large number of studies have found that spectral rsEEG can be a useful tool for auxiliary diagnosis of Alzheimer’s disease, post-stroke aphasia, and post-stroke depression. However, few studies have used rsEEG as a VSN screening tool. Previous studies have found that resting EEG topography has high sensitivity and reliability, and can help distinguish patients with different severities of VSN (Pirondini et al., 2020). However, there are no studies on the spectral power parameters of rsEEG.

The delta/alpha ratio (DAR) and pairwise-derived brain symmetry index (pdBSI) are commonly used resting-state EEG parameters, which are potentially valuable early predictors of the severity of post-stroke dysfunction (Saes et al., 2020, 2021). Hemispheric stroke has been associated with increased low-frequency oscillations in delta bands (Cassidy et al., 2020) and decreased alpha activity (Zappasodi et al., 2019). Compared to the individual spectral components, the DAR quantifying these spectral power changes may more sensitively reflect the severity of neurological deficits. The pdBSI assesses the asymmetry of the spectral power distribution between hemispheres after unilateral hemispheric stroke by calculating the power spectral densities along with homologous EEG channel pairs (Sheorajpanday et al., 2009). However, it is still unknown whether these rsEEG parameters differ in post-stroke VSN patients compared to healthy subjects and patients without VSN after stroke, and whether they are related to the severity of VSN.

In this study, we aimed to determine the specific rsEEG characteristics in poststroke patients with visuospatial neglect. The rsEEG of patients with VSN after stroke, patients without VSN after stroke and healthy individuals were collected, and the differences in rsEEG parameters (DAR and pdBSI) and topographic maps among the three groups and their correlation with clinical manifestations were analyzed. We aimed to determine whether specific rsEEG features of these post-stroke VSN patients could be used to aid in diagnosis and evaluation and to help design clinical screening procedures for visuospatial neglect in post-stroke patients.

## Materials and methods

### Participants

Patients were recruited from sequential admissions to the Department of Rehabilitation at the Xuanwu Hospital of Capital Medical University, China. A total of 44 first-event subacute stroke patients after right hemisphere damage were included, comprising of 26 patients with left VSN and 18 patients without VSN (non-VSN). The demographic characteristics are reported in Tables 1, 2. We also recruited 18 age-matched healthy controls (HC). The inclusion criteria for stroke patients were as follows: (1) first-ever cerebral stroke according to computed tomography or magnetic resonance imaging (MRI) scan; (2) ability to complete the necessary checks; (3) age ≥ 18 years; and (4) all right-handed patients who had normal or corrected-to-normal visual acuity. The exclusion criteria were as follows: (1) other neurological diseases; (2) severe cognitive problems, that is, Mini-Mental State Examination score < 18; and (3) worsening condition. All participants provided written informed consent and the study was approved by the Ethics Committee of Xuanwu Hospital (approval number: [2020]155).

**TABLE 1:** Stroke characteristics.

Subject number	Gender	Age (years)	Duration after stroke (days)	Type of stroke	Lesion location
VSN01	Female	47	16	Ischemic	T, P, BG
VSN02	Male	48	31	Ischemic	BG
VSN03	Male	62	9	Ischemic	F, BG
VSN04	Male	61	16	Ischemic	F, T, P, BG, CS
VSN05	Male	67	21	Ischemic	F, T, BG
VSN06	Female	69	18	Ischemic	F, T, P, BG, CR
VSN07	Male	69	25	Ischemic	F, T, P, CS
VSN08	Female	66	12	Ischemic	F, T
VSN09	Female	51	18	Ischemic	F, T, P, BG
VSN10	Male	58	36	Ischemic	F, T, P
VSN11	Female	73	19	Ischemic	F, T, P
VSN12	Male	48	17	Hemorrhagic	BG
VSN13	Male	37	42	Hemorrhagic	F, T, P
VSN14	Male	58	17	Ischemic	BG
VSN15	Male	65	13	Ischemic	F, BG
VSN16	Male	61	30	Hemorrhagic	BG
VSN17	Female	31	24	Ischemic	F, T, P, BG
VSN18	Female	71	10	Ischemic	F, BG
VSN19	Female	78	23	Ischemic	F, T, P, BG
VSN20	Male	49	22	Ischemic	F, T, P, BG, CR
VSN21	Male	59	14	Ischemic	F, T, P, BG
VSN22	Female	66	24	Ischemic	BG, CS
VSN23	Male	67	28	Ischemic	F, P, CR
VSN24	Male	73	8	Ischemic	T, BG
VSN25	Male	56	40	Ischemic	F, T, BG
VSN26	Male	67	17	Hemorrhagic	P
Non-VSN01	Male	74	21	Ischemic	BG
Non-VSN02	Male	45	14	Hemorrhagic	F, BG
Non-VSN03	Male	53	15	Ischemic	F, T, P, BG, CR
Non-VSN04	Male	38	19	Ischemic	BG
Non-VSN05	Male	69	14	Ischemic	F, P, BG
Non-VSN06	Male	53	8	Ischemic	BG
Non-VSN07	Male	43	26	Hemorrhagic	F, T, P, BG
Non-VSN08	Female	63	41	Ischemic	BG, CR
Non-VSN09	Male	65	20	Ischemic	F, T, P, CR
Non-VSN10	Female	69	22	Ischemic	F, T, P, BG
Non-VSN11	Male	58	27	Ischemic	BG
Non-VSN12	Male	72	20	Ischemic	BG, CR
Non-VSN13	Male	63	32	Hemorrhagic	F, P, BG
Non-VSN14	Female	66	10	Ischemic	BG
Non-VSN15	Female	68	15	Ischemic	T, P, BG, CS
Non-VSN16	Male	68	21	Ischemic	F, P, BG
Non-VSN17	Male	62	20	Ischemic	F, P
Non-VSN18	Male	57	18	Hemorrhagic	F, T, P, BG

BG, Basal ganglia; CR, Corona radiata; CS, Centrum semiovale; F, Frontal lobe; T, Temporal lobe; P, Parietal lobe.

**TABLE 2:** Patients’ demographic and clinical characteristics.

	VSN (*n* = 26)	Non-VSN (*n* = 18)	HC (*n* = 18)	*p*
Gender(female/male)	9/17	4/14	5/13	0.666
Age(*M* ± SD)	59.88±11.40	60.33±10.35	54.72±9.10	0.197
Type of stroke (ischemic/hemorrhagic)	22/4	14/4	–	0.857
Lesion location (cortical/cortico-subcortical/subcortical)	1/21/4	1/10/7	–	0.185
Poststroke time(*M*± SD; Day)	21.15±9.04	20.17±7.85	–	0.709
BI	34.23±15.28	53.06±19.41	–	0.001
FMA	30.27±18.84	50.11±16.47	–	0.004
BBS	8.19±8.20	16.39±13.55	–	0.016

All stroke patients were assessed for activities of daily living (ADL), motor function, and balance. The Barthel index (BI) was used to assess patients’ daily living abilities. The total BI score was 100, and higher scores suggest stronger daily living ability. The Fugl-Meyer Motor Assessment (FMA) Scale included upper and lower extremity movements, with 33 assessment items for upper extremity movement and 17 assessment items for lower extremity movement, with a total score of 100. Higher scores indicated better limb motor function. The Berg Balance Scale (BBS) was a commonly used balance scale that can comprehensively evaluate the dynamic and static balance function of stroke patients in the sitting and standing positions. It consisted of 14 items with a total score of 56. A lower score indicates poorer balance function.

### Clinical assessment of visuospatial neglect

#### Line bisection task

The patients were asked to bisect five horizontal black lines of differing lengths (80, 100, 120, 140, and 160 mm). The deviation of the patient’s marked point from the true midpoint of the line (in millimeters) was measured and converted to a percentage score (line bisection error [LBE]). Rightward deviations from the true line center were scored as positive and leftward deviations were scored as negative. VSN was diagnosed when the average LBE was > 12%.

#### Line cancellation task

This test involved 30 lines, each with a length of 15 mm, evenly distributed on the paper. Stroke patients were asked to cross all the lines on the page. The ratio of the number of missing line segments to 30 was the omission rate for the line cancellation task (ORL).

#### Clock copying task

The subjects were instructed to copy a clock on paper. Errors of omission in hands and numbers were considered pathological VSN.

#### Star cancellation task

There were 56 small stars interspersed with 52 large stars, 10 short words, and 13 letters. All patients were asked to mark all small stars. The omission rate of stars (ORS) was the number of missing small stars divided by 56.

### Electroencephalography

All subjects were requested to relax and not engage in any specific mental activity during EEG recording. Eye-closed rsEEG signals were recorded for 5 min using a NeuroScan NuAmps amplifier (Compumedics United States, Ltd., El Paso, TX, United States), and 64 Ag–AgCl electrodes were mounted on a Quik Cap using a modified 10–20 placement scheme to record the EEG. The EEG data were recorded with a 0.1–100 Hz band-pass filter at a sampling rate of 1,000 Hz. The ground electrode was placed on the forehead and the reference electrode was placed on the nose. The impedance of all the electrodes was maintained at ≤ 10 kΩ.

#### Pre-processing

Offline EEG preprocessing was conducted using the open-source EEGLAB toolbox and custom MATLAB 2013b (Math Works, Natick, NA). The raw EEG data were filtered using an FIR filter at 0.1–40 Hz. A 48–52 Hz notch filter was used to eliminate the power frequency interference. The data were then segmented into 2 s epochs. Bad channels were discarded by visual inspection and interpolated using the spherical method, followed by re-referencing to the remaining average. Data portions contaminated by eye blinks and eye movements were corrected using independent component analysis (ICA). The EEG epochs with amplitude values exceeding ± 100 μV at any electrode were excluded. The power of spectral density (PSD, μV^2^/Hz) using fast Fourier transform (FFT) was carried out for four frequency bands: delta (1–4 Hz), theta (4–8 Hz), alpha (8–13 Hz), and beta (13–30 Hz).

#### Resting-state electroencephalography parameters

##### Delta/alpha ratio

The DAR was defined as the ratio between the mean delta power and mean alpha power. For every channel *c*, the power of the delta and alpha (*f* = 1,…,4 Hz and *f* = 8,…,12 Hz, respectively) was determined as the mean of the spectral power [*P_*c*_(f)*]over this range. The DAR was computed as

$${\mathrm{DAR}}_{c}=\frac{{\left\langle P_{c}\,\left(f\right)\right\rangle }_{f=1,\ldots ,4\,\mathrm{Hz}}}{\langle P_{c}{\left(f\right)}_{f=8,\ldots ,12\,\mathrm{Hz}}}$$

The ratios were averaged over all N EEG channels yielding the global DAR as:

$$\mathrm{DAR}=\frac{1}{N}\,\sum_{c=1}^{N}{\mathrm{DAR}}_{c}$$

In addition, the DAR was calculated over the affected hemisphere (DAR_AH_) and the unaffected hemisphere (DAR_UH_), excluding the electrodes covering the midline.

##### Pairwise-derived brain symmetry index

The pdBSI was defined as the absolute pairwise normalized difference in spectral power between the homologous channels *C*_*L*_ and *C*_*R*_ for the left and right hemispheres, excluding the electrodes covering the midline. The difference was averaged over a range of 1–25 Hz, according to

$${\mathrm{BSI}}_{\mathrm{CP}}=\left\langle \left|\frac{P_{C_{R}}\,\left(f\right)-P_{C_{L}}\,\left(f\right)}{P_{C_{R}}\,\left(f\right)+P_{C_{L}}\,\left(f\right)}\right|\right\rangle \,f=1,\ldots ,25\,\mathrm{Hz}$$

These values were averaged over all channel pairs (cp):

$$\mathrm{BSI}=\frac{2}{N}\,\sum_{\mathrm{CP}=1}^{N/2}{\mathrm{BSI}}_{\mathrm{CP}}$$

The pdBSI estimated the global asymmetry along with homologous channel pairs, ranging from 0 to 1, with 0 defined as the maximal symmetry. In addition, the BSI values of each frequency band and of the alpha frequency band of the parieto-occipital region (Pz, P1, P2, P3, P4, P5, P6, P7, P8, POz, PO3, PO4, PO5, PO6, PO7, PO8, Oz, O1, O2) were also calculated.

### Statistical analysis

All statistical analyses were performed using IBM SPSS Statistics V22.0 (IBM Corp, Armonk, NY, United States). The distribution of data was tested for normality using the Kolmogorov–Smirnov test. Categorical data were analyzed using the chi-squared test. One-way ANOVA with *post hoc* testing using Bonferroni’s test was used to test the differences among the three groups (VSN, non-VSN, and HC). Differences between the VSN and non-VSN groups were compared using an independent sample *t*-test. Pearson correlation analysis was performed to investigate the relationship between rsEEG parameters and patient characteristics. In addition, EEG parameters that showed a significant difference among groups were analyzed using receiver operating characteristic (ROC) curve analysis. The sensitivity/specificity cut-off values, positive predictive value (PPV), and negative predictive value (NPV) were determined using Youden’s index.

## Results

### Demographic information and descriptive data

The relevant demographic and clinical characteristics of the three groups were shown in Table 2. There were no significant differences in age or sex among the three groups. No significant difference was found in the type of stroke or time since stroke onset between VSN and non-VSN subjects. The BI, FMA, and BBS scores of the VSN patients were significantly lower than those of the non-VSN patients.

**FIGURE 1:**
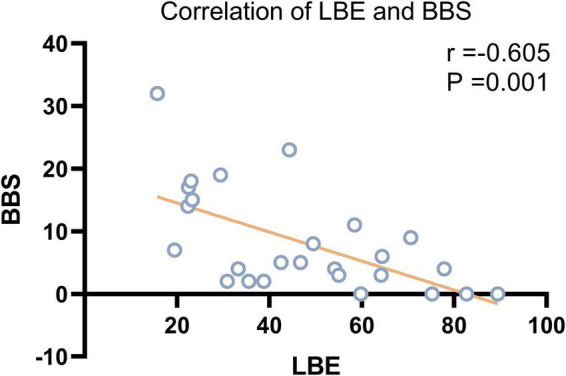
Correlation between LBE and BBS.

According to the Pearson correlation analysis results, LBE scores were significantly correlated with BBS scores (*r* = –0.605, *P* = 0.001) but not with BI or FMA scores in patients with VSN. Scatter plots showing the relationship between LBE and BBS are presented in Figure 1.

### Electroencephalography parameters

#### Delta/alpha ratio

**FIGURE 2:**
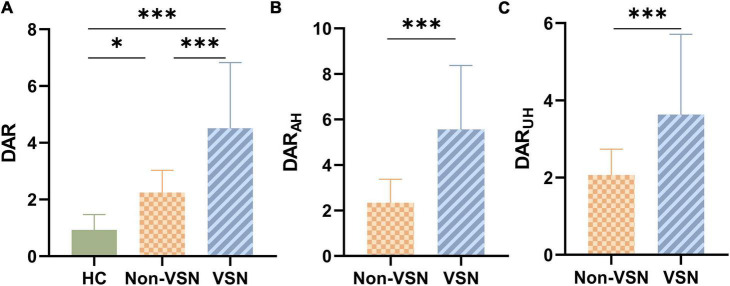
DAR values for each group. **(A)** DAR values between three groups. **(B)** DAR_AH_ values between Non-VSN and VSN. **(C)** DAR_UH_ values between Non-VSN and VSN.*p < 0.05; ***p < 0.001.

The ANOVA results shown in Figure 2 indicated that there were significant differences in the DAR among the three groups. DAR values were higher in patients with VSN compared with both patients without VSN and HCs (*F* = 28.348, *P* < 0.001; VSN vs HC: *P* < 0.001; VSN vs Non-VSN: *P* < 0.001). Patients with non-VSN had a higher DAR than HCs (*P* = 0.048). Patients with VSN had higher DAR_AH_ and DAR_UH_ values than non-VSN patients.

**FIGURE 3:**
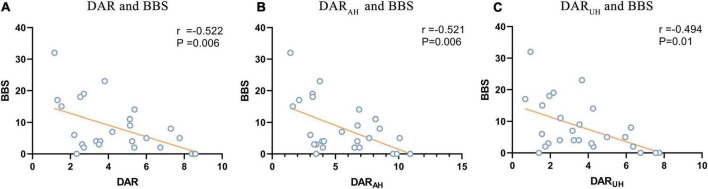
Signifcant correlations between DAR values and BBS. **(A)** Correlation between DAR and BBS. **(B)** Correlation between DAR_AH_ and BBS. **(C)** Correlation between DAR_UH_ and BBS.

For the DAR, a trend toward a negative association with BBS was found in patients with VSN, as were DAR_AH_ and DAR_UH_ (see Figure 3; DAR: *r* = –0.522, *P* = 0.006; DAR_AH_: *r* = –0.521, *P* = 0.006; DAR_UH_: *r* = –0.494, *P* = 0.01). In patients with VSN, no significant correlation was found between the DAR, DAR_AH_, DAR_UH_, BI, or FMA. In patients without VSN, no significant correlation was found between the DAR and BI, FMA, or BBS.

#### Pairwise-derived brain symmetry index

**FIGURE 4:**
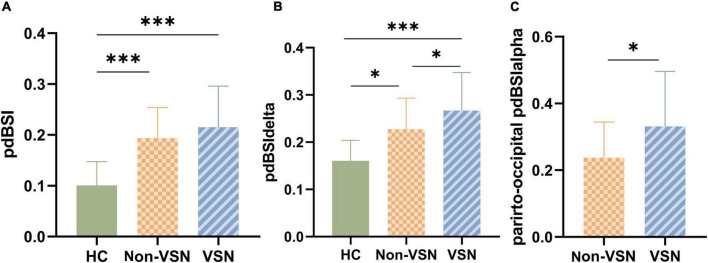
pdBSI values for each group. **(A)** pdBSI values between three groups. **(B)** pdBSIdelta values between three groups. **(C)** Parieto-occipital pdBSIalpha values between Non-VSN and VSN.*p < 0.05; ***p < 0.001.

As shown in Figure 4, pdBSI values were significantly elevated in both non-VSN and VSN patients compared with healthy subjects (*F* = 16.822, *P* < 0.001; VSN vs HC: *P* < 0.001; Non-VSN vs HC: *P* < 0.001). These differences were most pronounced in the delta and theta bands. We found no significant difference in pdBSI values between patients with and without VSN. However, there were significant differences in pdBSIdelta and parieto-occipital pdBSIalpha between patients with and without VSN patients.

**FIGURE 5:**
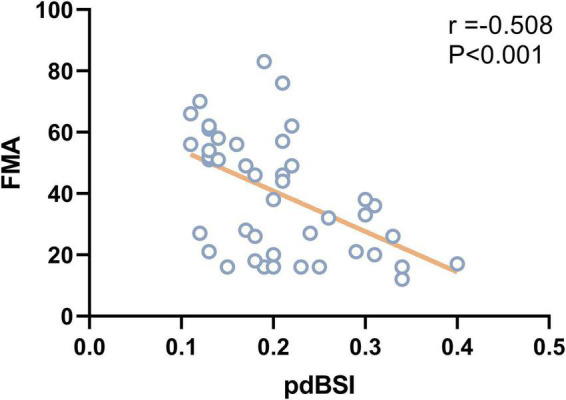
Correlation between LBE and BBS.

As shown in Figure 5, pdBSI showed a trend toward a negative association with FMA in stroke patients (*r* = –0.508, *P* < 0.001), mainly in the delta and theta frequency bands. No correlation was found between pdBSI and BI or BBS in both VSN and non-VSN patients.

#### Topographic plots

**FIGURE 6:**
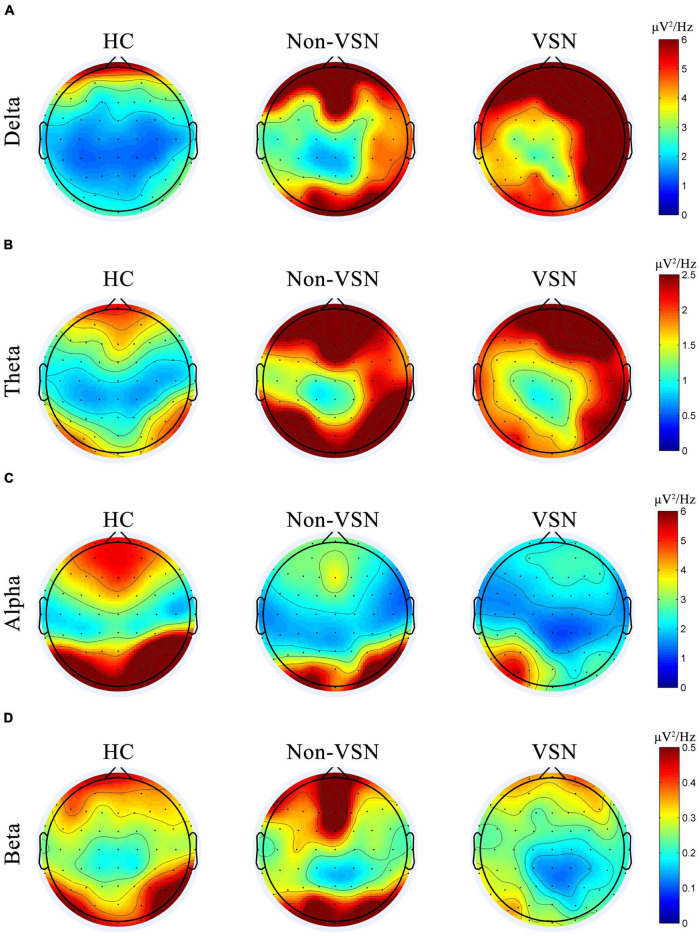
Topographic plots of the delta **(A)**, theta **(B)**, alpha **(C)**, and beta **(D)** frequency bands in three groups.

We found increased delta and theta power and decreased alpha and beta power of the EEG in VSN subjects. Regarding delta power, significant abnormalities were found in the right frontal, parietal and temporal areas in VSN patients compared to HCs, but they were located only in the right parietal areas compared to non-VSN patients. For theta power, the significantly abnormal brain regions in patients with VSN were mainly the frontal and parietal areas compared to non-VSN patients. For alpha power, significant abnormalities were found in the frontal, parietal, and occipital regions of patients with VSN compared with HCs, but they were located only in the right parietal and occipital areas compared with non-VSN patients. The topographic plots of the delta, theta, alpha, and beta frequency bands of the groups are shown in Figure 6.

### Association between electroencephalography parameters and symptom of visuospatial neglect

**FIGURE 7:**
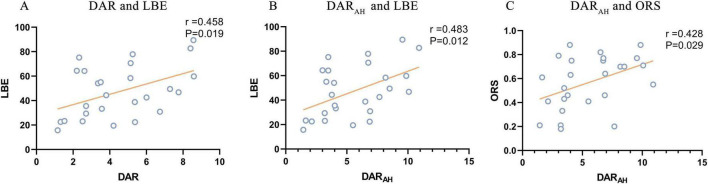
Significant correlations between DAR values and paper-pencil test. **(A)** Correlation between DAR and BBS. **(B)** Correlation between DAR_AH_ and BBS. **(C)** Correlation between DAR_UH_ and BBS.

**FIGURE 8:**
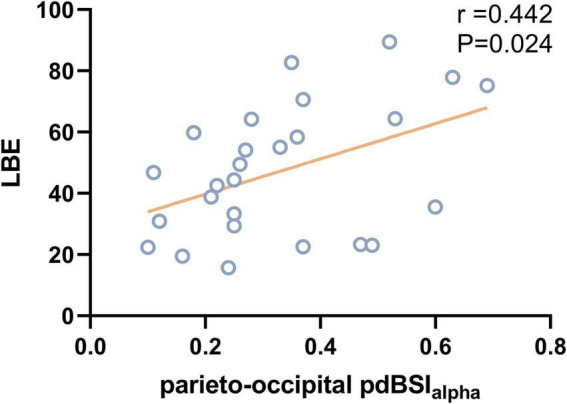
Correlation between parieto-occipital pdBSIalpha and LBE.

The DAR showed a trend toward a positive association with LBE (*r* = 0.458, *P* = 0.019). Furthermore, the DAR_AH_ showed a positive correlation with LBE (*r* = 0.483, *P* = 0.012) and ORS (*r* = 0.428, *P* = 0.029; see Figure 7). No correlation was found between the DAR and ORL. Parieto-occipital pdBSI_alpha_ showed a trend toward a positive association with the LBE (*r* = 0.442, *P* = 0.024), but no correlation was found with the ORL or ORS (see Figure 8). There was no significant correlation between the pdBSI in the other frequency bands and the paper-pencil test.

### Receiver operating characteristic analysis for diagnostic discrimination

The area under the ROC curve (AUC), cutoff value, sensitivity, specificity, PPV, and NPV of each EEG parameter for differentiating between non-VSN patients and HCs were shown in Table 3. The results of ROC analyses showed that the DAR (AUC = 0.870, cut off = 1.778, *P* < 0.001), pdBSI (AUC = 0.867, cut off = 0.118, *P* < 0.001) and pdBSI_delta_ (AUC = 0.728, cut off = 0.201, *P* = 0.02) could discriminate non-VSN patients and HCs. The sensitivity of the pdBSI was the highest (0.90); however, the specificity was much lower (0.63). The specificity of the DAR was the highest (0.99), while the sensitivity was 0.71.

**TABLE 3:** Results from receiver operating characteristic (ROC) analysis to distinguish non-VSN from HC.

EEG measure	AUC [95% CI]	Cutoff value	Sensitivity	Specificity	PPV (%)	NPV (%)
DAR	0.870 [0.753, 0.987]	1.778	0.63	0.99	98.44	72.90
pdBSI	0.867 [0.751, 0.983]	0.118	0.90	0.71	75.27	87.05
pdBSI_delta_	0.728 [0.562, 0.893]	0.201	0.53	0.88	81.68	65.04

AUC, area under the receiver operating curve; CI, confidence interval; PPV, positive predictive value; NPV, negative predictive value.

As shown in Table 4, the ROC analysis indicated that the DAR (AUC = 0.803, cut off = 3.472, *P* = 0.001), DAR_AH_ (AUC = 0.865, cut off = 3.112, *P* < 0.001), and DAR_UH_ (AUC = 0.731, cut off = 3.141, *P* = 0.01) could discriminate between patients with and without VSN. The pdBSI_delta_ and parieto-occipital pdBSI_alpha_ had poor diagnostic ability. The sensitivity of DAR_AH_ was 0.85, and the specificity was 0.78. The specificities of DAR and DAR_UH_ were high (0.94 and 0.99, respectively); however, the sensitivities were generally much lower (0.62 and 0.58, respectively).

**TABLE 4:** Results from receiver operating characteristic (ROC) analysis to distinguish VSN from non-VSN.

EEG measure	AUC [95% CI]	Cutoff value	Sensitivity	Specificity	PPV (%)	NPV (%)
DAR	0.803 [0.674, 0.933]	3.472	0.62	0.94	91.65	71.03
DAR_AH_	0.865 [0.760, 0.971]	3.112	0.85	0.78	79.21	83.48
DAR_UH_	0.731 [0.581, 0.880]	3.141	0.58	0.99	98.29	70.06
pdBSI_delta_	0.688 [0.530, 0.846]	0.252	0.62	0.78	73.48	66.90
parieto-occipital pdBSI_alpha_	0.658 [0.495, 0.821]	0.212	0.81	0.50	61.77	72.25

AUC, area under the receiver operating curve; CI, confidence interval; PPV, positive predictive value; NPV, negative predictive value.

## Discussion

In this study, we analyzed the resting-state EEG of three groups (patients with VSN, patients without VSN, and HCs) using spectral analysis. We found that patients with VSN performed poorly compared with those without VSN. Resting-state EEG has high temporal resolution, sensitivity, and specificity for evaluating the severity of VSN. Spectral analysis of the rsEEG revealed increased DAR, DAR_AH_, DAR_UH_ and parieto-occipital pdBSI_alpha_ in stroke patients with VSN, and these parameters measure the severity of VSN and are reliable markers of VSN after stroke. The DAR_AH_ was shown to be highly sensitive to VSN and showed a good ability to discriminate poststroke patients with VSN. It has the potential to assist in the identification of VSN after stroke and is useful for clinical rehabilitation.

VSN has profound implications for quality of life after stroke; however, there is a lack of consensus regarding the screening and diagnosis of this syndrome due to the heterogeneity of its clinical manifestations. In routine stroke unit assessment, VSN was greatly underdiagnosed with a missed diagnosis rate of up to 56% (Puig-Pijoan et al., 2018). The line bisection task, line cancellation task, and star cancellation task were shown to be more sensitive tests for diagnosing VSN (Azouvi et al., 2006). However, these paper-and-pencil tests have some limitations. When ceiling and floor effects cause the scale to not truly reflect the severity of VSN, the recording of patients’ electrical brain activity may be more sensitive. Because many stroke patients cannot complete task-state EEG owing to factors such as reduced cognitive level and fatigue, resting-state EEG is more suitable for VSN patients after stroke. Previous studies in healthy participants and stroke patients have shown that quantitative parameters of resting-state brain activity, such as the spectral power of different bands, are intra-individually stable in repeated measurements (Dalton et al., 2021; Duan et al., 2021). The characterization of resting-state brain activity is a reliable biomarker that may aid in clinical decision-making and treatment selection (Saes et al., 2019; Sebastian-Romagosa et al., 2020). In the current study, we explored whether spontaneous brain activity could be used as a diagnostic and assessment tool in poststroke patients with VSN.

Higher delta and theta activity in the right fronto-parietal region and lower alpha activity in the right parieto-occipital region were found in patients with VSN. Compared with HCs, DAR values were higher in both VSN and non-VSN patients; most importantly, they were significantly higher in VSN patients than in non-VSN patients. Furthermore, the DAR and DAR_AH_ were positively correlated with the paper-pencil test scale. This reveals that the DAR contains unique information regarding visuospatial neglect impairments. Excessive delta power after stroke is associated with cognitive function. Delta frequencies may reflect alertness modulation involving the corticothalamic and corticocortical neural circuits (Rabiller et al., 2015). Alpha oscillations are considered markers of vigilance, attention, cognitive processing, and cortical communication in both healthy individuals and patients (Sadaghiani and Kleinschmidt, 2016; Clayton et al., 2018). Resting EEG studies of healthy people found that alpha power in the right hemisphere was greater than that in the left hemisphere (Cicek et al., 2003). This greater right hemisphere EEG alpha activity may explain the prominent role of the right hemisphere in attention. In stroke patients with VSN, however, we found a reduction in alpha power in the right parieto-occipital region. This suggests that alpha neural oscillations may underlie the electrophysiological underpinnings of widespread attentional network connectivity in both hemispheres. VSN was initially thought to be a parietal syndrome; however, an increasing number of functional magnetic resonance imaging and EEG studies have confirmed that VSN is a disturbance in the attention network (Corbetta and Shulman, 2011; Ros et al., 2022). The parietal lobe, particularly the posterior parietal cortex (PPC), is a critical component of the attentional network. The bilateral parietal lobes compete to mediate direct attention to the contralateral space. The posterior rsEEG alpha in healthy individuals was associated with LBT performance (Cicek et al., 2003). Task EEG studies in healthy humans have shown a relative reduction in alpha-band activity in the parieto-occipital hemisphere contralateral to the direction of spatial attention, possibly reflecting enhanced cortical excitability (Banerjee et al., 2011). In contrast, patients with VSN show pathologically enhanced alpha oscillations during both baseline fixation and cue orientation when completing a spatial orientation task (Lasaponara et al., 2019). This pathological enhancement was significantly associated with the severity of VSN and damage to white matter fiber tracts. Transcranial magnetic stimulation, inhibits cortical activity in the right PPC, disrupts attentional processes, affects visuospatial attention, and induces transient spatial neglect-like symptoms in healthy adults (Fierro et al., 2000; Mariner et al., 2021). Moreover, multiple studies have demonstrated that transcranial magnetic stimulation of the PPC can effectively improve the symptoms of patients with VSN after stroke (Salazar et al., 2018; Ye et al., 2021). Therefore, alpha oscillations in the parietal cortex are a reliable biomarker of visuospatial neglect after stroke and may be useful for rehabilitation interventions involving non-invasive brain stimulation and EEG-based neurofeedback.

Not only did DAR values increase in the affected hemisphere after stroke compared to healthy individuals, but DAR values also increased in the unaffected hemisphere. Although structural damage from stroke is focal, remote dysfunction may occur in areas of the brain that are distant from the damaged area (Siegel et al., 2016). This view of distributed brain network connectivity disturbances provides new insights into the recovery from post-stroke dysfunction. The bimodal balance recovery theory suggests that the recovery of dysfunction in some stroke patients may be related to the contralateral hemisphere (Di Pino et al., 2014), and this has been confirmed in patients with visuospatial neglect (Cao et al., 2016). In fact, the DAR_AH_ was higher than the DAR_UH_ in some patients with VSN, and the opposite was observed in other patients in our study. Whether this inconsistency is related to the patient’s recovery pattern is unclear and further research is warranted.

Analyses of the pdBSI index indicated no significant hemispheric asymmetry in healthy participants. In contrast, patients with damage to the right hemisphere showed significantly increased low-frequency oscillations in the lesional hemisphere. Our results showed that patients with subacute right hemisphere stroke had significantly higher pdBSI values than healthy subjects. Higher BSI values reflect a greater power asymmetry in the hemispheres. This finding is consistent with those of other studies (Sheorajpanday et al., 2011). However, we found no significant difference in pdBSI values between patients with and without VSN. Indeed, previous studies have found that pdBSI is significantly associated with infarct volume after controlling for various confounding factors (Sheorajpanday et al., 2011). The interhemispheric asymmetry represented by pdBSI persisted during the chronic phase of stroke (Saes et al., 2019). In healthy individuals, the bilateral hemispheres competitively inhibit each other to achieve balance, but this balance is broken after a stroke. The inhibitory effect of the contralateral hemisphere on the affected hemisphere was higher, which results in interhemispheric asymmetry. Interhemispheric asymmetry can affect recovery from dysfunction. Our study found that higher pdBSI values, mainly pdBSI_delta_ and pdBSI_theta_, but not DAR, were associated with lower FMA in patients with stroke with or without VSN, which is consistent with the results of some studies (Saes et al., 2019). However, other studies have come to the opposite conclusion that compared with differences between cerebral hemispheres, DAR values can be more sensitive in assessing the severity of dyskinesia in stroke patients (Brito et al., 2021). The low number of EEG channels (nine scalp electrodes) used in the study by Brito et al. may have contributed to these inconsistent results. The slower delta and theta frequencies are thought to be generated by cortical layers II-VI. Low-frequency cortical activity may reflect the integrity of the cortical-cortical network connectivity. Previous studies also found that higher BSI_theta_ values were significantly negatively correlated with upper extremity motor function 6 months after stroke (Saes et al., 2020, 2021). Therefore, low-frequency oscillations may reflect both injury and recovery after stroke and may be a reliable biomarker for stroke rehabilitation (Cassidy et al., 2020). Our study found that visuospatial neglect following stroke affects the EEG alpha rhythm, mainly in the right parietal and occipital areas. This resulted in marked asymmetry of the alpha band in the parietal and occipital regions, which was significantly associated with neglect severity. These results showed that interhemispheric asymmetry in the alpha band of the parieto-occipital region can provide a measure of the severity of neglect.

Thus, rsEEG may be a useful tool for identifying patients with VSN after stroke. In the present study, we found that the use of the DAR_AH_ to distinguish between patients with VSN and non-VSN patients was more than 80% sensitive and 70% specific. Therefore, DAR_AH_ is a promising marker for the diagnosis of VSN after stroke. This finding is important for the early diagnosis of VSN after stroke. In distinguishing non-VSN patients from healthy individuals, DAR is an indicator with high specificity and low sensitivity, while pdBSI is an indicator with high sensitivity and low specificity. MRI is contraindicated in some patients and expensive. In contrast, EEG may allow an inexpensive, reliable bedside evaluation with practically no contraindications. Due to the advantages of EEG, there have been many studies on EEG-assisted diagnosis of acute stroke (Erani et al., 2020; van Meenen et al., 2021). The low sensitivity of the DAR in this study was inconsistent with that of previous studies. Studies have found that a DAR of 3.7 has 100% sensitivity and 100% specificity in distinguishing acute ischemic stroke from healthy individuals (Finnigan et al., 2016). This study collected patients in the acute phase, while our study collected patients in the subacute phase. The spectral signature of rsEEG changes over time, especially in the delta frequency band. Differences in EEG acquisition time and processing methods may have contributed to the differences in the studies. Therefore, to facilitate the use of rsEEG for stroke diagnosis, standardized EEG acquisition and processing procedures are required. In addition to this, larger sample size and other methods, such as coherence, should also be used to assess the accuracy of rsEEG aids in diagnosis.

Some studies found that VSN patients were more dependent on ADL than non-VSN patients (Bosma et al., 2020), a finding that is consistent with our results. VSN can disrupt a patient’s balance and affect motor function recovery. Compared with the line bisection and star cancellation tasks, no correlation was found between the line cancellation task and the resting-state EEG parameters. This may be because the line cancellation task contained only 30 line segments, and the calculated omission rate was not as sensitive as that of the line bisection task or star cancellation task.

The current study had several limitations. First, the sample size of the current study is insufficient, which weakens the influence of the article; however, we will conduct further studies with a larger sample size to confirm the feasibility of the research conclusions. Second, we only assessed patients at admission and did not follow up with these patients. Although our study demonstrated that resting-state EEG may be a useful tool for identifying potential VSN after stroke, EEG parameters can also reflect the severity of VSN after stroke. However, over time, the spectral features show a gradual normalization. Studies have found that the DAR value of patients with chronic stroke is not different from that of healthy people. We do not know whether rsEEG remains a useful assessment tool for VSN after spontaneous recovery in patients with VSN. A longitudinal assessment of patients with stroke was not performed, and the relationship between EEG parameters and VSN could not be clearly defined. Future studies should expand the single time point to multiple time points to verify our conclusions further. Third, stroke was divided into acute, subacute, and chronic phases, but this study only included patients in the subacute phase; therefore, further research is needed to perform subgroup analysis on acute and chronic phase patients. Fourth, due to lack of MRI data, this study only corrected for lesion location without calculating lesion volume. However, it did not calculate the lesion area in stroke patients.

## Conclusion

To the best of our knowledge, this is the first study on VSN resting-state EEG spectral analysis after stroke. Overall, our EEG results are consistent with those of previous EEG studies and provide new evidence for rsEEG features of visuospatial neglect after stroke. The rsEEG cortical asymmetry and the DAR were increased in patients with visuospatial neglect after stroke. Higher asymmetry in the parieto-occipital region of the alpha band and higher DAR values are associated with more severe visuospatial neglect. Furthermore, our study showed that resting-state DARAH can accurately differentiate between patients with and without VSN after stroke. This implies that rsEEG could be used for the auxiliary diagnosis of VSN after stroke, and the DAR and pdBSI alpha in resting EEG may be useful biomarkers of visuospatial neglect after stroke.

## Data availability statement

The original contributions presented in the study are included in the article, further inquiries can be directed to the corresponding author.

## Ethics statement

The studies involving human participants were reviewed and approved by Ethics Committee of Xuanwu Hospital. The patients/participants provided their written informed consent to participate in this study.

## Author contributions

YZ: drafting and revising the manuscript, study concept and design, analysis and interpretation of data. LY: study concept and design, acquisition of data, revising the manuscript, and obtaining funds. LC: study concept and design, and revising the manuscript. WS: study concept and design, analysis and interpretation of data, and study supervision. All authors contributed to the article and approved the submitted version.
